# Efficacy of BETTER transitional care intervention for diverse patients with traumatic brain injury and their families: Study protocol of a randomized controlled trial

**DOI:** 10.1371/journal.pone.0296083

**Published:** 2024-02-23

**Authors:** Tolu O. Oyesanya, Stephanie O. Ibemere, HyunBin You, Maralis Mercado Emerson, Wei Pan, Anushka Palipana, Melissa Kandel, Darius Ingram, Mayra Soto, Anne Pioppo, Brittany Albert, Tamia Walker-Atwater, Jodi Hawes, Jordan Komisarow, Katherine Ramos, Lindsey Byom, Rosa Gonzalez-Guarda, Courtney H. Van Houtven, Suresh Agarwal, Janet Prvu Bettger

**Affiliations:** 1 Duke University School of Nursing, Durham, NC, United States of America; 2 Duke Global Health Institute, Durham, NC, United States of America; 3 Duke University School of Medicine, Durham, NC, United States of America; 4 Department of Rehabilitation Services, Duke University Health System, Durham, NC, United States of America; 5 Department of Allied Health Sciences, University of North Carolina at Chapel Hill, Chapel Hill, NC, United States of America; 6 Durham VA Health Care System, Center of Innovation to Accelerate Discovery and Practice Transformation (ADAPT), Durham, NC, United States of America; 7 Department of Health and Rehabilitation Sciences, Temple University, Philadelphia, PA, United States of America; PLOS: Public Library of Science, UNITED KINGDOM

## Abstract

**Objective:**

The purpose of this study is to examine the efficacy of BETTER (Brain Injury, Education, Training, and Therapy to Enhance Recovery) vs. usual transitional care management among diverse adults with traumatic brain injury (TBI) discharged home from acute hospital care and families.

**Methods:**

This will be a single-site, two-arm, randomized controlled trial (N = 436 people, 218 patient/family dyads, 109 dyads per arm) of BETTER, a culturally- and linguistically-tailored, patient- and family-centered, TBI transitional care intervention for adult patients with TBI and families. Skilled clinical interventionists will follow a manualized protocol to address patient/family needs. The interventionists will co-establish goals with participants; coordinate post-hospital care, services, and resources; and provide patient/family education and training on self- and family-management and coping skills for 16 weeks following hospital discharge. English- and Spanish-speaking adult patients with mild-to-severe TBI who are discharged directly home from the hospital without inpatient rehabilitation or transfer to other settings (community discharge) and associated family caregivers are eligible and will be randomized to treatment or usual transitional care management. We will use intention-to-treat analysis to determine if patients receiving BETTER have a higher quality of life (primary outcome, SF-36) at 16-weeks post-hospital discharge than those receiving usual transitional care management. We will conduct a descriptive, qualitative study with 45 dyads randomized to BETTER, using semi-structured interviews, to capture perspectives on barriers and facilitators to participation. Data will be analyzed using conventional content analysis. Finally, we will conduct a cost/budget impact analysis, evaluating differences in intervention costs and healthcare costs by arm.

**Discussion:**

Findings will guide our team in designing a future, multi-site trial to disseminate and implement BETTER into clinical practice to enhance the standard of care for adults with TBI and families. The new knowledge generated will drive advancements in health equity among diverse adults with TBI and families.

**Trial registration:**

NCT05929833.

## Introduction

Each year, more than 2.7 million U.S. people of all ages sustain a TBI, with higher incidence among adults and among racial/ethnic minoritized populations. Though limited data are available, U.S. race-specific annual incidence per 100,000 for TBIs evaluated in emergency departments reported 569 for Black, 457 for White, and 345 for other minoritized populations.[1] The effects of TBI have short- and long-term disproportionate effects on U.S. Black and Latino communities, represented by racial/ethnic inequities in post-acute outcomes.[2] Up to 1 year after discharge home, Black and Latino patients with TBI have worse physical, mental, and social outcomes than their White counterparts,[3] evidenced by higher rates of rehospitalizations,[4] long-term disability,[5] depression,[6] substance abuse,[7] and unemployment.[5, 8] These outcomes continue to have tremendous, chronic implications for nuclear and extended family members of Black and Latino patients,[3, 9] especially for patients who were in school or working, may not yet be financially independent, have small children, and/or still require support from parents.[10] As the percentage of U.S. racial/ethnic minoritized populations are predicted to reach 45% of the population in the next 30 years, it is imperative that the health of racial/ethnic minorities with TBI be adequately addressed to advance health equity.[11]

The complexity of TBI-related impairments, combined with the fragmentation of healthcare services, creates the perfect storm for low patient quality of life (QOL), mismanaged symptoms, rehospitalizations, and increased caregiver strain.[12, 13] Lack of insurance or access to care, as well as language barriers, aggravate these issues.[14] Despite complex health needs, there are no U.S. clinical standards for transitional care management for any patients, including patients with TBI.[12] Transitional care is defined as actions in the clinical encounter designed to ensure the coordination and continuity of care for patients transferring between different locations or levels of care.[15] Poor transitions are a result of inadequate planning, insufficient patient/family education and training, and limited and fragmented access to essential services.[15] These gaps are often compounded by lack of insurance,[15] social services and supports, and language barriers.[16]

For other patients with acute events (e.g., stroke, myocardial infarction), transitional care interventions have been shown to improve patient QOL and health outcomes with strategies like individualized transitional care plans, post-discharge care coordination, and community-based referrals.[15, 17, 18] Yet, the few existing TBI transitional care interventions do not: 1) show efficacy for Black and Latino patients; 2) have cultural or linguistic tailoring to meet needs/preferences of diverse patients/families; 3) focus on behavioral change, or 3) address family needs.[19–22] The early post-acute period (within 16 weeks of hospital discharge to home) includes high rates of unmet patient/family needs and preventable clinical events,[23] making this timeframe an ideal point for interventions to guide improvements in health and QOL for adults with TBI and families.[15] Yet, the current state of usual transitional care management often leaves patients with TBI and their families to independently navigate and access fragmented services and supports.[13, 24–29] It is critical that TBI transitional care evolves to meet the needs of adults with TBI and their families with attention to specific needs of racial/ethnic minorities to drive equitable advancements in care for minoritized communities.

The prevailing racial/ethnic inequities in TBI outcomes and the paucity of theory-driven, evidence-based TBI transitional care interventions led our team to develop a culturally- and linguistically-tailored intervention named **BETTER** (**B**rain Injury, **E**ducation, **T**raining, and **T**herapy to **E**nhance **R**ecovery).[30–32] We developed and refined BETTER with our findings from mixed-methods research with key stakeholders to identify needs and areas to improve in the TBI transitional care process.[13, 15, 24–27, 33–49] We built capacity to enroll Spanish-speaking patients and families via cultural and linguistic adaptation of the intervention and accompanying materials.[30]

The objective of this randomized controlled trial (RCT) is to examine the efficacy of **BETTER** [30–32] (vs. usual transitional care management) among diverse adults with TBI and families. The primary hypothesis is that adults with TBI receiving BETTER will have higher QOL at 16-weeks post-hospital discharge than those receiving usual transitional care management.

The specific aims of this study are:

1. To examine the efficacy of BETTER vs. usual transitional care management on patients’ QOL at 16-weeks post-hospital discharge among dyads of diverse adults with TBI and families.
2. To explore barriers and facilitators to patient/family participation in the BETTER TBI transitional care intervention.
3. To perform a cost and budget impact analysis of the BETTER TBI transitional care intervention.

The purpose of this manuscript is to describe the protocol for the BETTER randomized controlled trial.

## Methods

### Study design

**Fig 1:**
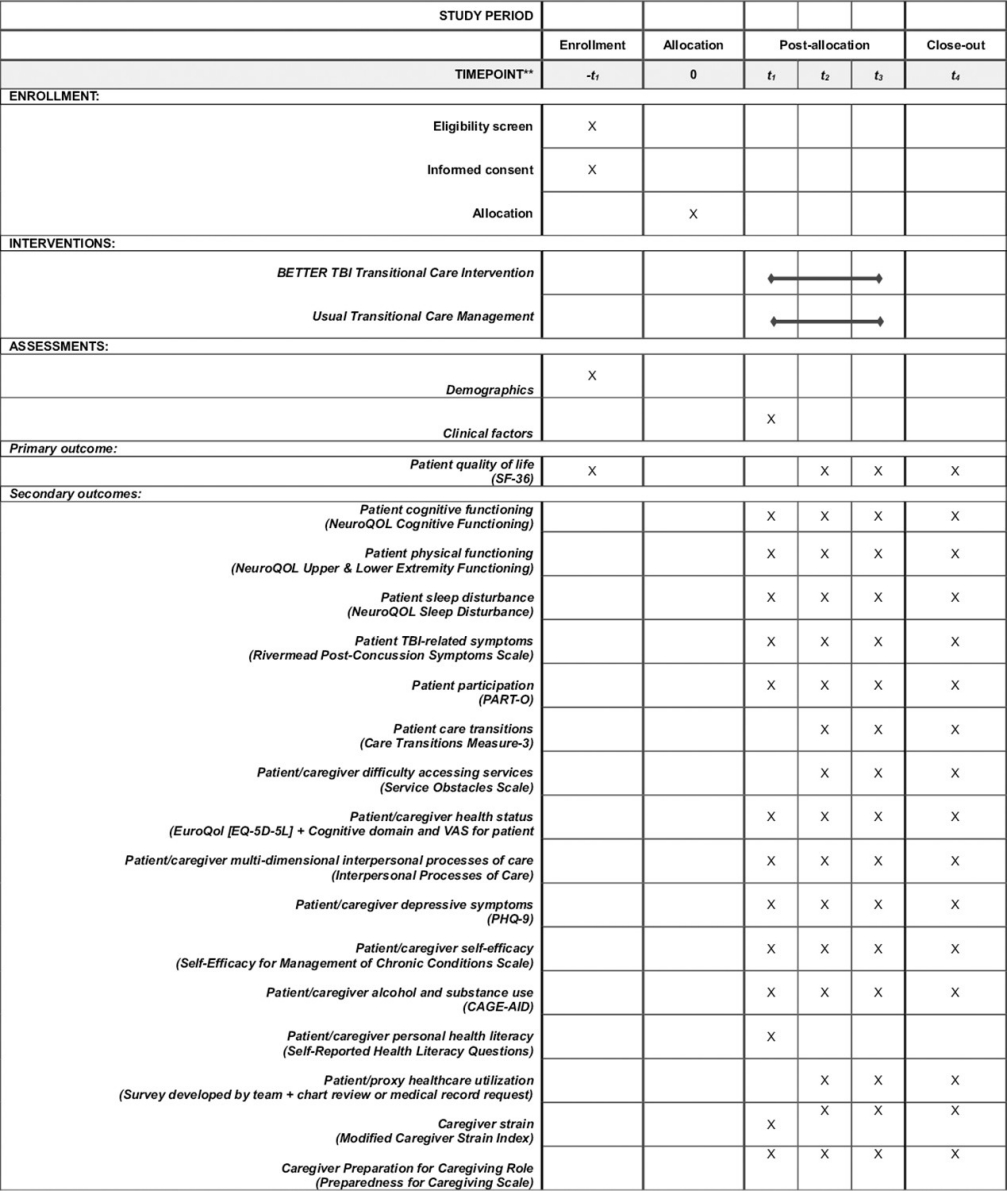
SPIRIT schedule of enrollment, intervention, and assessments.

**Fig 2:**
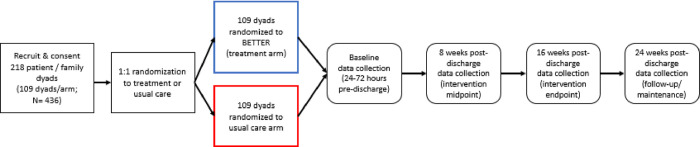
Randomized controlled trial study design.

This will be a single-site, two-arm, randomized controlled trial of BETTER, a culturally- and linguistically-tailored, patient- and family-centered, behavioral, TBI transitional care intervention for diverse English- and Spanish-speaking patients with TBI and their families, compared to usual transitional care management. In addition to baseline assessments at 24–72 hours pre-hospital discharge for both groups, follow-up assessments will occur at 8-weeks, 16-weeks, and 24-weeks post-discharge from acute hospital care to home. Dyads in both the treatment and usual transitional care management arms will complete the same data collection measures at the same time points to allow for comparison in the longitudinal analyses (see **Fig 1** for SPIRIT schedule of enrollment, intervention, and assessments). The study design of the BETTER RCT is illustrated in detail (see **Fig 2** for RCT design). The RCT is registered with clinicaltrials.gov (NCT05929833) and is funded by the National Institutes of Health (Grant # R01NR020818).

### Setting

Recruitment will occur at a Level I trauma centered located in the southeastern U.S. Recruitment is set to begin January 2024.

### Sample

Patients with TBI of any race/ethnicity, regardless of insurance status, will be eligible if they are: a) age ≥18 years; b) diagnosed with mild, moderate, or severe TBI [admission Glasgow Coma Scale score of 3–15];[50] c) admitted to an inpatient acute care unit; d) to be discharged directly home from the acute hospital without inpatient rehabilitation or transfer to other settings (community discharge); e) sufficient cognitive functioning to participate (i.e., able to follow 2-step commands), as determined by the Galveston Orientation and Amnesia Test (score ≥76 eligible);[51] f) English- or Spanish-speaking (self-report), and g) access to a phone or computer with internet capabilities for study participation. Patients with TBI will be excluded if they have: a) pre-injury cognitive impairments, early dementia, or Alzheimer’s disease, b) acute, unstable neurologic condition(s), c) severe psychiatric diagnosis that is untreated with no psychiatric provider on record, d) been admitted from settings or locations other than home, or e) not been able to identify a family member to participate with them in the study.

Family members will include patient-identified biological relatives and friends [52] and are eligible if they: a) are an anticipated primary caregiver after discharge (i.e., plans to live in same home as patient *or* have direct contact with patient ≥10 hours/week);[53] b) age ≥18 years; c) English- or Spanish-speaking (self-report), and d) access to a phone or computer with internet capabilities for study participation. Family members will be excluded if the associated patient is not eligible or declines participation. All participants must be able to consent to participate. Patients and their associated family caregiver will be recruited.

### Intervention description

The intervention was informed by our team’s TBI-related mixed methods research;[13, 24–27, 30, 31, 33–43, 45, 46, 54–58] literature used to support, educate, and train patients and families in recovery;[59–62] as well as the Individual and Family Self-Management Theory.[63] BETTER is a culturally and linguistically tailored, patient- and family-centered, behavioral intervention with the aim of improving patients’ QOL (SF-36 total score, primary outcome) at 16-weeks following hospital discharge. Trained clinical interventionists address patient/family dyad’s needs using co-established goals; coordinated post-hospital care, services, and resources; and patient/family education on self- and family-management and coping skills ≤16 weeks post-hospital discharge. BETTER is delivered 24–72 hours pre-hospital discharge until 16-weeks post-hospital discharge, with ≥1 weekly contact with each patient/family dyad from an assigned clinical interventionist. BETTER is remotely delivered, allowing for convenient engagement of participants in the COVID-19 pandemic and for scalability.

**Fig 3:**
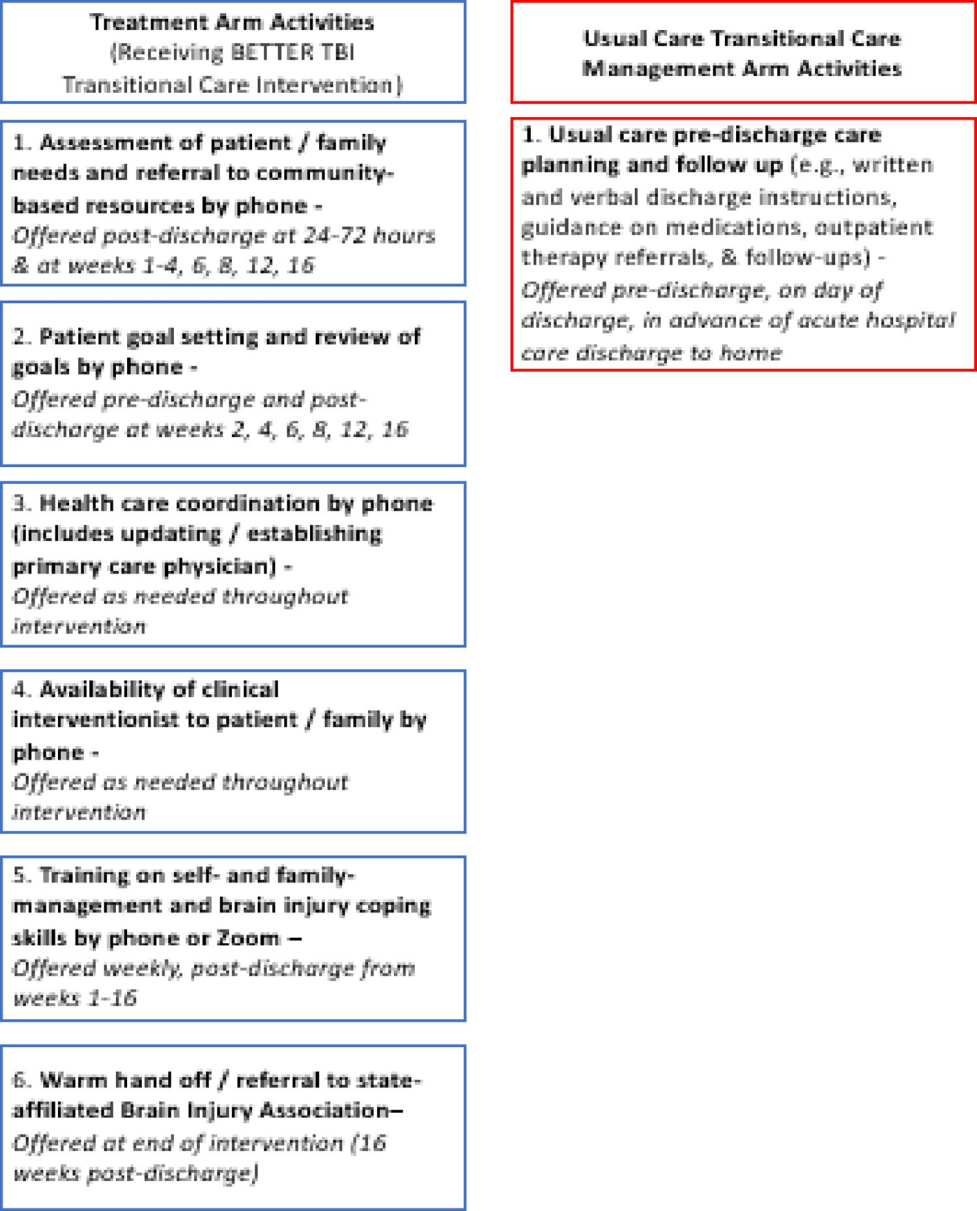
Treatment vs. usual transitional care management arm activities.

The six components of BETTER include (**Fig 3**): 1) assessment of patient/family needs and referral to community-based resources; 2) patient goal setting and review of goals; 3) health care coordination; 4) availability of clinical interventionist to patient/family; 5) training on self- and family-management and brain injury coping skills; and 6) warm hand off/referral to the state-affiliated Brain Injury Association at the end of the intervention for additional resources. At the beginning of the study, materials distributed to patient/family dyads include a pillbox for the patient and two printed participant workbooks, one for the patient and one for the caregiver. Detailed guidance for delivering BETTER, including a directory of community-based resources to refer dyads to that are free or subsidized for insured and uninsured participants, as well as training for interventionists, are outlined in our manualized intervention protocol. Additional information on sources used to inform the development of BETTER are published elsewhere.[30–32]

We assessed the feasibility of BETTER in a prospective, quasi-experimental, single-arm, single-center pilot study.[31, 32] Clinical interventionists were two occupational therapists. Recruitment occurred from February to July 2021. Data were collected from adults with TBI (age 18–64 years) with mild, moderate, and severe TBI, who were discharged home from acute hospital care and family caregivers (N = 31).[31, 32] Findings showed BETTER significantly improved patients’ physical QOL (SF-36 [64]) by 31.36 points (p = 0.006) from 24–72 hours pre-hospital discharge (baseline) to 16-weeks post-hospital discharge (intervention endpoint), and that BETTER was feasible and acceptable with adults with TBI and families.[30–32] There were no significant differences in clinical outcomes by race/ethnicity.[31, 32] Feasibility study findings were used to inform the design of this RCT.

#### Interventionist training

As occurred in the feasibility study, clinical interventionists will be occupational therapists with ≥3 years of clinical experience with neurological or trauma care. Occupational therapists are uniquely qualified to serve as interventionists for BETTER, as they are aptly trained to address occupational performance and contexts in patients with TBI, such as issues with executive functioning, instrumental activities of daily living, and patient-centered goal setting. Research has shown that occupational therapists’ use of cognitive behavioral strategies, goal-directed interventions, and functional skills training after TBI can result in improvements in patient QOL. [59, 65]. We will hire and train at least 4 interventionists, and ≥1 will be bilingual (English- and Spanish-speaking). One interventionist from the feasibility study has been invited to participate in this study and will be asked to oversee the work of the other interventionists in conjunction with supervision from the PI and select co-investigators. Interventionists must complete our robust training protocol, which includes up to 65 hours of training and role-playing on the protocol in advance of recruitment/enrollment; they will also engage in intervention fidelity strategies throughout the study, as well as ongoing supervision with the PI and co-investigators for the study duration.

#### Intervention fidelity

Strategies to ensure intervention fidelity will include: 1) interventionists audio/video recording and documenting all contacts/sessions with dyads, 2) documenting all intervention components delivered to each dyad on our intervention fidelity checklist (yes/no) to ensure interventionist adherence to all protocol activities, and 3) weekly supervision between interventionists, PI and select co-investigators. Based on our feasibility study, our anticipated intervention fidelity rate is 98%.

### Usual transitional care management

In alignment with U.S. usual transitional care management for patients with TBI, usual transitional care management arm activities for adults with TBI and their family caregivers (see **Fig 3**) already include usual transitional care management discharge planning process and follow-up (e.g., verbal and written discharge instructions, with guidance on medications, outpatient therapy, and follow-up appointments) [13, 24]. U.S. usual transitional care management for patients with TBI does not typically consist of any intervention activities planned for treatment arm participants, such as assignment to work with a clinical interventionist, needs assessment, resource referral, goal setting, care coordination, or training on self- and family-management and brain injury coping skills. However, depending on age or other clinical factors, a small number of patients (e.g., older adults) may qualify for resource referral and care coordination by phone.

### Measures

Detailed list of data collection measures and timeframes of data collection are outlined in Table 1. Our team has secured the existing Spanish versions of data collection measures. Data collection measures not available in Spanish have been culturally and linguistically translated from English to Spanish (forward and back translation), and we obtained and incorporated feedback from native Spanish speakers into our translations. Details on our translation process are published elsewhere [44].

**Table 1:** Data to be collected & time points for treatment & usual transitional care management arm dyads of adults with TBI and families (Aim 1).

		Administered to		Data collection times			
				Pre-	Post-discharge		
Measures / Type of Data to Be Collected	Description / Construct of focus	Patient	Family	Pre-	8 wks	16 wks	24 wks
Demographics	Age, sex, race/ethnicity, education, pre-injury occupation, insurance status, annual income	X	X	X			
Patient clinical factors (via chart review)	TBI severity, length of stay, comorbidities	X		X			
^+^Short Form-36 (SF-36)—primary outcome	Quality of life (QOL); SF-36 is commonly used to assess QOL in patients with TBI	X		X	X	X	X
NeuroQOL, Cognitive functioning	Cognitive functioning status	X		X	X	X	X
NeuroQOL, Upper & Lower extremity functioning (separate measures)	Physical functioning status	X		X	X	X	X
NeuroQOL, Sleep Disturbance	Sleep disturbances	X		X	X	X	X
Rivermead Post-concussion Questionnaire (RPQ)	TBI-related symptoms	X		X	X	X	X
PART-O	Participation	X			X	X	X
Care Transitions Measure-3 (CTM-3)	Process of care transitions	X			X	X	X
Service Obstacles Scale (SOS)	Difficulty in accessing services	X	X		X	X	X
EuroQol (EQ-5D-5L) + VAS (visual analogue scale) + Cognitive domain (C)	Health status, including five dimensions (i.e., mobility, self-care, usual activities, pain/discomfort, anxiety/depression) and the VAS, with the cognitive domain (C) added for patients only	X	X	X	X	X	X
Interpersonal Processes of Care (IPC)	Multi-dimensional interpersonal processes in the clinical encounter	X	X	X	X	X	X
*Patient Health Questionnaire-9 (PHQ-9)	Depressive symptoms	X	X	X	X	X	X
Self-Efficacy for Management of Chronic Conditions Scale	Confidence in chronic TBI management	X	X	X	X	X	X
CAGE-AID Substance Abuse Screening Tool	Alcohol and substance use	X	X	X	X	X	X
Self-Reported Health Literacy Questions	Personal health literacy	X	X	X			
Health Care Utilization (patient and proxy/family-report, confirmed via chart review or medical record request)	Health services used by 16 weeks post-discharge, e.g., follow-ups, therapy, emergency department/urgent care, rehospitalizations	X	X		X	X	X
Modified Caregiver Strain Index	Caregiver strain		X	X	X	X	X
Preparedness for Caregiving Scale	Preparation for caregiver role		X	X	X	X	X

Note: ^+^ = SF-36 is primary outcome. wks = weeks. ED = emergency department * = If an individual expresses suicidal ideation (via PHQ-9), we will implement our escalation protocol & create a formal safety plan with them. Details on plans for addressing suicidal ideation and other adverse events are listed in our human subjects plan & data safety monitoring plan section of the manualized intervention protocol.

### Primary outcome measure

Patient-reported QOL, measured by SF-36,[64] will be the primary outcome of this trial. The SF-36 is commonly used to assess QOL in patients with TBI [66]. The SF-36 has two subscales, physical (i.e., physical functioning, role limitations due to physical health, pain, general health) and mental component summaries (i.e., role limitations due to emotional health, vitality, mental health, social functioning) with a total of eight health concepts [64]. SF-36 scores are weighted sums of the items in each health concept. Scores of each health concept range from 0 to 100, with higher scores representing better quality of life.

#### Secondary outcomes

Secondary outcomes for the patient include cognitive and physical functioning;[67] sleep disturbance;[67] TBI-related symptoms;[68] participation;[69] and process of care transitions.[70] Secondary outcomes for the patient and caregiver include difficulty accessing services;[71] health status;[72] multi-dimensional interpersonal processes in the clinical encounter;[73] depressive symptoms;[74] self-efficacy;[75] alcohol and substance use;[76] personal health literacy;[77, 78] and healthcare utilization. Secondary outcomes for the caregiver only will include caregiver strain[79] and preparedness for caregiving.[80]

### Patient secondary outcomes

Patient cognitive and physical functioning and sleep disturbance will be measured by NeuroQOL cognitive functioning, upper extremity functioning, lower extremity functioning, and sleep disturbance short forms.[67] Each NeuroQOL short form measure consists of 8-items, scores ranging from 8 to 40, where higher scores indicate higher functioning or more sleep disturbance, respectively.[67] TBI-related symptoms will be measured by the Rivermead Post-concussion Symptoms Questionnaire (RPQ), which is a 16-item scale.[68] Scores on the first 3 items of the RPQ can range from 0 to 12, with higher scores indicating higher severity of early symptom clusters of post-concussive symptoms; scores on items 4 to 16 can range from 0 to 52, with higher scores indicating higher severity of later post-concussive symptoms.[68] Participation will be measured using the Participation Assessment with Recombined Tools-Objective (PART-O), an objective measure of societal participation developed for patients with TBI with 24-items, with higher scores indicating higher levels of participation.[69] The process of care transitions will be measured by the Care Transitions Measure-3, a 3-item scale with scores ranging from 0 to 100, with higher scores indicating less challenges with the transitional care process.[70]

### Patient/caregiver secondary outcomes

Difficulty accessing services will be measured by the Service Obstacles Scale, a 6-item scale with scores ranging from 7 to 42, with higher scores indicating more difficulty accessing services.[71] Health status will be measured by the EuroQol, the EQ-5D-5L, which has been found to be responsive and dynamic over time for persons with TBI.[72] The EuroQol consists of five health domains, including mobility, self-care, usual activities, pain/discomfort, and anxiety/depression, where each domain is scored on a 1 to 5 scale, with higher scores indicating higher levels of perceived problems in the domain. Patients with TBI will also complete the cognitive domain of the EuroQol (EQ-5D-5L+C), to capture condition specific issues; we will also use the VAS to record the patient and caregiver’s self-rated health on a vertical visual analogue scale [81, 82] Multi-dimensional interpersonal processes in the clinical encounter, particularly discrimination in the clinical encounter, will be measured using the Interpersonal Processes of Care survey (short-form).[73] The measure, which contains 18-items, has 7 domains using a 1 to 5 scale, with scores ranging from 18 to 90. Higher scores indicate a higher frequency of interpersonal challenges and discrimination in the clinical encounter. Depressive symptoms will be assessed by the Patient Health Questionnaire-9 (PHQ-9), which is a 9-item scale, with scores ranging from 0 to 27, where higher scores indicate a greater level of depression.[74] Self-efficacy will be measured by the Self-Efficacy for Managing Chronic Conditions Scale, which is a 6-item scale, with scores ranging from 0 to10, where higher scores indicate higher level of self-efficacy.[75] Alcohol and substance use will be measured using the CAGE-AID Substance Use Screening Tool, a 5-item measure with scores ranging from 0 to 4, with higher scores indicating higher possibility of substance use disorder and need for possible testing.[76] Health literacy will be measured using the Self-Reported Health Literacy Questions, a 3-item measure with scores ranging from 3–15, with higher scores indicating lower self-reported health literacy.[77, 78] For health care utilization, we will use a survey developed by our team, which captures the healthcare services accessed by the patient (e.g., emergency department, urgent care, therapy, primary or specialty care provider) after discharge; this survey will be completed by the patient and their proxy (family caregiver). This information will be confirmed via chart review or medical record request

### Caregiver secondary outcomes

Caregiver strain will be measured by the Modified Caregiver Strain Index, a 13-item scale, with scores ranging from 0 to 26, where higher scores indicate a higher level of caregiver strain.[79] Caregiver preparedness will be measured by the Preparedness for Caregiving Scale, a 8-item scale, with scores ranging from 0 to 32, where higher scores indicate the caregiver feels more prepared for the caregiving role.[80]

### Demographic and clinical factors

In addition to the above listed measures, we will also collect data on demographics and clinical factors. Demographics will include age, sex, race/ethnicity, education, pre-injury occupation, insurance status, and annual income. Clinical factors (via chart review) will include TBI severity, length of stay, and comorbidities.

### Data collection

For aim 1, a trained, bilingual (English- and Spanish-speaking) research coordinator will collect all longitudinal data from treatment and usual transitional care management arm patient/family dyads in-person or by phone at the following 4 timepoints: 1) 24–72 hours pre-hospital discharge (baseline); 2) 8-weeks post-hospital discharge (this is intervention midpoint for treatment arm dyads); 3) 16-weeks post-hospital discharge (this is intervention endpoint for treatment arm dyads); and 4) 24-weeks post-hospital discharge (follow-up/maintenance timepoint for treatment arm dyads). The 24-week follow-up/maintenance timepoint will be used to assess short-term benefits maintained after completion of the intervention. We will use all outcome data collected to assess efficacy (Aim 1) of our primary outcome, patient QOL (change in SF-36 total score[64]), for our intervention, as well as for analysis of secondary outcomes. Dyads in both the treatment and usual transitional care management arms will complete the same data collection measures at the same time points to allow for comparison in the longitudinal analyses (see **Fig 1** for SPIRIT schedule of enrollment, intervention, and assessments).

For recruitment rates, the research coordinator will record the number of patient/family dyads eligible, approached, and consented to participate. Based on our feasibility study, our anticipated recruitment is ≥50% of participants approached to participate. For enrollment rates, the research coordinator will record the number of patient/family dyads recruited and enrolled into the intervention. Based on our feasibility study, our anticipated enrollment is ≥90% of participants recruited. For data collection rates, the research coordinator will record for each follow-up data collection timepoint: 1) number of patient/family dyads called and reached; 2) number of call attempts per patient/family dyad; 3) number of patient/family dyads reached but unable to provide information; 4) length of phone call; 5) day of week and time of day patient/family dyads participated; and 6) completeness of data collected. The research coordinator will obtain baseline data by in-person or by phone from dyads 24–72 hours pre-hospital discharge. At 8-, 16-, and 24-weeks post-hospital discharge, the research coordinator will call patient/family dyads to obtain follow-up data. Based on our feasibility study, our anticipated data collection rate at baseline is 100% of patient/family dyads; our anticipated data collection rate for 8- and 16-weeks post-hospital discharge is 80% of dyads. Finally, our anticipated data collection rate at 24-weeks post-hospital discharge is 65% of dyads. We proposed more conservative estimates for recruitment, enrollment, and data collection than rates seen in our feasibility study.[31, 32] Our team will review recruitment and follow-up rates weekly and identify strategies to improve recruitment, enrollment, and data collection. For participant engagement rates, in accordance with the protocol, the interventionists will document each study activity delivered to each dyad and the length of time it took to complete each activity, useful for determining intervention intensity. Based on our feasibility study, our anticipated participant engagement rate is 75% of all intervention activities.

For aim 2, semi-structured interviews with BETTER dyads will be conducted by phone to assess participants’ perspectives on participation; interviews will be conducted by a trained, bilingual (English- and Spanish-speaking) research coordinator. We will select 45 treatment patient/family dyads (N = 90) who received BETTER to provide perspectives on barriers and facilitators to participating in BETTER. We will sample dyads stratified by three participation levels/interviewee groups: 15 dyads who complete ≤25% of intervention activities, 15 who complete 50–75%, and 15 who complete >75%. This sample size is guided by NIH Stage Model for Intervention Development guidelines of including 15–30 participants per interviewee group.[83] Interview questions will be on advantages of participating in BETTER, what participants valued or perceived to be most helpful, as well as concerns, challenges, and disadvantages of participating in BETTER. The interview guide will be available in English and Spanish and will be piloted before use. Each private interview will take approximately 10 minutes, and will be audio recorded and transcribed and translated verbatim by the research coordinator.

For aim 3, the average cost of delivering the intervention per dyad will be quantified by arm along with the average total costs of transitional care costs (post-discharge health service use) reported over 16-weeks post-discharge by arm, and will be tested for differences between arms. BETTER’s total transitional care costs (inclusive of post-discharge health service use) will be calculated as these costs are expected to increase given the intervention activities regarding care coordination and promoting appropriate referrals to meet needs. The cost analysis will be conducted regardless of effectiveness findings. If BETTER is effective at increasing QOL, a budget impact analysis will be conducted to inform the feasibility of scaling BETTER at a health system level. This approach will consider intervention costs and total health care costs with a priori sensitivity analyses as described below. It will also incorporate the average EuroQol scores by arm, as an additional measure of QOL. Whereas not powered for a full cost-effectiveness analysis, descriptively evaluating the EuroQol will allow comparison to other trials supporting adults with TBI.

Intervention-related costs will be assessed using a micro-costing approach and will be detailed by intervention components. Intervention-related costs include labor costs. Capital costs, which will be minimal beyond office space and are fixed costs that would not change with a larger implementation of BETTER, will be omitted. Labor costs will include: 1) time of investigators to train the interventionists, 2) research coordinator time spent recruiting/enrolling study participants, and 3) interventionist time spent being trained for and delivering intervention activities. Hourly wage and fringe benefit rates will be applied to the time spent per task to derive total labor costs, assessed using salaries and study data. Other intervention costs (e.g., software modification, patient recruitment) will be based on specific personnel’s annual salary plus benefits. Interventionist time spent conducting the intervention will be tracked by study interventionists. Time spent by research team members (i.e., research staff, interventionist) conducting research activities, but that would not necessarily be incurred if BETTER were implemented in clinical practice, will be tracked as well, so these costs can be excluded from intervention costs. Total intervention costs will be divided by the number of patients in each study arm to derive per-patient intervention cost, though individual patient costs may vary by intervention intensity.

### Ethical considerations

Ethical approval has been obtained from the Duke University Health System Institutional Review Board (Pro00112309), including approval for electronic, written consent, with plans for protection of confidentiality discussed.

### Recruitment, screening, and enrollment

Recruitment for this study will occur 24–72 hours before acute hospital care discharge to home. A trained, bilingual (English and Spanish-speaking) research coordinator will review the electronic health record daily to determine if eligible patients have been admitted to the hospital. The research coordinator will contact eligible patients in-person during the hospital stay or via phone to determine interest in participating and to explain the informed consent, clearly discuss the study purpose and planned activities, answer all questions, and conduct further eligibility screening for patients. Next, each patient with TBI will be asked to identify which family member they would like to participate in the study with them. The research coordinator will then contact the family member identified by the patient. If the family member is also interested, the research coordinator will explain the informed consent, clearly discuss the study purpose and planned activities, and answer all questions. Finally, the research coordinator will obtain a signed, electronic informed consent from both the patient and family member and will email each a copy for their own records. The primary threat to attrition in this study is loss to follow-up.[84] To reduce and prevent attrition, participants will each receive up to $250 ($50/data collection timepoint x 4 quantitative data collection timepoints [treatment & usual transitional care management arms] + $50 for qualitative interview [treatment arm only]) during the study period.

### Randomization

After written consent is obtained, patient/family dyads will be randomly assigned to one of the two study arms, the: (1) intervention or (2) usual transitional care management arm. Dyads will be randomly assigned to one of the two study arms, with stratification based on TBI severity. Due to the nature of the intervention, the participants, interventionists, and the investigators responsible for overseeing the BETTER intervention cannot be blind to the allocation of participants to each group.

### Data analysis

For Aim 1, a descriptive analysis will be initially performed by examining distributional properties of demographic and clinical variables both for the overall sample size, as well as stratified by whether the individual received BETTER or usual transitional care management. Similarly, data will be stratified by (1) primary language spoken, (2) racial/ethnic group, and (3) sex to ensure randomization was successful. Differences in categorical explanatory variables will be tested using chi-square techniques (or the Fisher Exact test, when appropriate); while differences in continuous explanatory variables will be tested using independent-samples t-tests (or ANOVA, when appropriate).

The effects of the two arms (treatment vs. usual transitional care management) on the primary outcome in this RCT will be estimated and compared using repeated measures ANOVA to support the central hypothesis that adults with TBI receiving BETTER will have higher QOL 16-weeks after hospital discharge than those receiving usual transitional care management. Similarly, for our secondary aims, we will further examine if such differences exist in SF-36 total scores between these groups, over time by (1) primary language spoken, (2) racial/ethnic group, and (3) sex. The primary analytic endpoint for Aim 1 is at 16-weeks post-discharge to assess the longevity and efficacy of our observed effects, consistent with previous literature.

The longitudinal design of this study also allows us to examine if effects are observed as early as 8-weeks post-discharge; and whether effects hold (or are further increased) at 16- and 24-weeks post-discharge. Thus, linear mixed-effects models (LMMs) will be conducted. The two arms (BETTER vs. usual transitional care management), will be analyzed as fixed effects and time will be analyzed as a repeated measures effect to examine whether these outcomes significantly change over time. Other main effects (primary language spoken, race/ethnicity, sex) will be incorporated as covariates during secondary analyses. All main effects and all interactions will be investigated for significance from the mixed-effects models.

For all the analyses, intent-to-treat approach will be adopted as pre-planned for main effect comparisons between (1) BETTER vs. usual transitional care management, (2) primary language spoken, (3) racial/ethnic group, and (4) sex. Also, the bootstrap interval estimates will be generated for comparisons in all the linear models when model assumptions are untenable.

For Aim 2, interview transcripts will be coded using conventional content analysis, a data analysis technique suitable in an area where little is known.[85] The qualitative team is well-versed in qualitative methods and will consist of three bilingual members. The team will first read through all translated transcripts to get a holistic sense of the data and write memos describing initial impressions.[85] The team will then independently code transcripts and meet to discuss and compare codes and reach consensus on discrepancies. Codes will be entered into a codebook, with code names and definitions and quotation exemplars. The codebook will evolve as coding continues. Similar codes will be grouped into themes.[85] Findings will be reported using thick description and quotation exemplars as evidence of findings. Several strategies will be utilized to increase trustworthiness and rigor of findings, including developing an audit trail, engaging in peer debriefing, and including detailed methods and context of our sample and data collection and analytical procedures in publications and presentations describing findings.[86]

For Aim 3, within each study arm, unadjusted and covariate-adjusted differences in intervention costs and health care costs by primary language spoken, racial/ethnic group, and sex will be evaluated. The primary analysis will be the unadjusted costs relying on randomization. To understand factors associated with any cost differences, secondary analysis will evaluate the association of patient characteristics with patient-level costs using a generalized linear model (SAS PROC GLM) with family and link function to fit the distribution of the outcome. If BETTER is effective at increasing QOL, budget impact will be evaluated by comparing the expected costs of system-wide intervention implementation. Expected costs will be estimated by calculating per patient incremental costs of the intervention and then multiplying by the number of eligible patients. Sensitivity analyses will assess the impact of varying the length, reach (that is, number of patients served out of total eligible patients expected), and timing of BETTER on fee-for-service (FFS) revenue eligibility. Probabilistic sensitivity analyses will be conducted using Monte Carlo simulations in Excel’s Crystal Ball.

### Sample size determination

Findings from the pilot study were combined with literature on the clinically meaningful difference in SF-36 total scores for patients with TBI between groups (0.3 standard deviations) to perform the power analysis for this study.[32, 87] For this study, a total of N = 356 individuals will provide 80% power for the study design described above (α = 0.05, two-tailed) to detect a small effect size between study arms (Cohen’s d = 0.18). A sample size of N = 356 represents 178 patient/family dyads with 89 dyads randomly assigned to the treatment arm and 89 dyads randomly assigned to the usual transitional care management arm. Based on our preliminary studies, we anticipate that 10% of eligible participants will decline participation and that 10% of recruited/enrolled participants will be lost to follow-up.[32] As such, to account for attrition, our enrollment goal is N = 436 individuals (218 patient/family dyads; 109 dyads per arm). It worth noting that the power calculations were performed only for the primary outcome, patient QOL changes in SF-36 total scores from baseline to 16 weeks post-hospital discharge, to test our central hypothesis. We powered this study to detect a small effect size to ensure we can detect the minimal clinically important difference.^31^

### Timeline

The timeline of this RCT is described in Table 2. Before starting, we obtained IRB approval. Aims will occur simultaneously and take 4 years and 9 months (6 months to hire and train interventionist; 3.25 years to recruit, enroll, and collect data; 1 year for analysis and dissemination). The timeline of this study was informed by our feasibility study.

**Table 2:** Timeline of the randomized controlled trial.

	Pre	Year 1				Year 2				Year 3				Year 4				Year 5		
Study Activities	Pre	Q1	Q2	Q3	Q4	Q1	Q2	Q3	Q4	Q1	Q2	Q3	Q4	Q1	Q2	Q3	Q4	Q1	Q2	Q3
Obtain IRB approval																				
Hire/train interventionists & research coordinators																				
Recruitment / enrollment																				
Data collection																				
Interventionist supervision																				
Data analyses (all aims)																				
Disseminate (publications & presentations)																				
Annual reporting																				

## Discussion

The objective of this RCT is to examine the efficacy of BETTER[30–32] (vs. usual transitional care management) among diverse adults with TBI and families. The proposed study aims to drive advancements in health equity through the inclusion of English- and Spanish-speaking participants, Black and Latino patients with TBI and family caregivers (who historically have poorer outcomes vs. Whites), and populations with limited or no access to care. This study also aims to improve systems and models of care by testing the efficacy of a new, TBI transitional care model.

Existing TBI-specific transitional care interventions have not been culturally or linguistically designed or adapted to address the needs/preferences of Spanish-speaking patients and families or allow for the inclusion of Spanish-speaking patients and families. To address this gap in research and practice, it is important for research and clinical teams to build the capacity to address the needs of Spanish-speaking patients with TBI and their families.[88] Access to Spanish-speaking professionals during the transitional care process, interpreters, and educational materials and content in Spanish is one important step in addressing transitional care needs of Spanish-speaking patients with TBI and their families.[89]

Findings from the trial will provide new knowledge to inform transitional care research for diverse adults with TBI and families and will generate evidence to improve public health and drive equitable advancements in care by reducing systemic and structural inequities in place for minoritized communities, with the goal of improving health equity.[90] This study’s findings will formally establish the efficacy of BETTER, informing future implementation and dissemination trials. Study findings may ultimately shift the standard of care for adults with TBI discharged home from acute hospital care and families and can inform the development of U.S. TBI transitional care standards.

## Supporting information

S1 Checklist (PDF)
